# Animalistic dehumanisation as a social influence strategy

**DOI:** 10.3389/fpsyg.2022.999959

**Published:** 2023-01-11

**Authors:** Alain Quiamzade, Fanny Lalot

**Affiliations:** ^1^Faculty of Psychology and Educational Sciences, University of Geneva, Geneva, Switzerland; ^2^Faculty of Psychology, UniDistance Suisse, Brig, Switzerland; ^3^Faculty of Psychology, University of Basel, Basel, Switzerland; ^4^School of Psychology, University of Kent, Canterbury, United Kingdom

**Keywords:** dehumanisation, animalisation, influence, resistance, essentialism, justification

## Abstract

The phenomenon of animalistic dehumanisation has been extensively studied in social psychology, but mostly as an intergroup relations tool used to justify the mistreatment of an outgroup. Surprisingly, however, dehumanisation has not been approached as an influence strategy to convince the ingroup to mistreat an outgroup. In the present article, we investigate these possible influence effects. We propose that a message depicting an outgroup in negative animalised terms would lead to lasting unfavourable outgroup attitudes because the animal essence conveyed through the message would immunise ingroup members against subsequent counterinfluence attempts. In one experimental study we compared the effect of three influence messages depicting a despised outgroup (Roma beggars) in negative animalised vs. negative humanised vs. positive humanised terms, followed by a counterpropaganda message advocating for Roma beggars’ rights. Results show that the animalisation message leads to a lasting animalised perception of the outgroup (eliciting disgust and repugnancy) that resists exposure to the counterpropaganda positive message. In contrast, the negative humanisation message provokes a brief negative perception of the group (pre-counterpropaganda) that disappears after exposure to the counterpropaganda. The animalisation message also leads to more negative attitudes and discriminatory behavioural intentions towards Roma beggars expressed after the counterpropaganda message (i.e., discrimination in the workplace, hiring intentions, and social proximity), whilst the negative humanisation message does not, showing no difference with the positive humanisation message. These results suggest that animalistic dehumanisation indeed acts as an influence strategy, immunising targets against subsequent counterpropaganda attempts. We discuss implications in the light of essentialisation, forms of dehumanisation and group status, and current non-discriminatory norms.

## 1. Introduction

### 1.1. Dehumanisation and its study in social psychology

Over the past decades, research in social psychology has extensively studied the phenomenon of dehumanisation, defined as the process of denying a human being proper humanness, notably: autonomy, individuality, and a sense of dignity (for reviews, see Haslam et al., 2007; Vaes et al., 2012; Haslam and Loughnan, 2014; Haslam and Stratemeyer, 2016; for a critic of the concept, see Lang, 2020). Haslam (2006) distinguishes two forms of dehumanisation based on two different senses of humanness. On the one hand, humanness may be considered as a set of features that are typical of humans. This sense of humanness leads to what Haslam calls “mechanistic dehumanisation”: stripping people from their human nature and seeing them, instead, as machines incapable of warmth, emotion, and individuality. On the other hand, humanness may be considered as the set of unique human characteristics that defines the boundary separating humans from other animals. This second sense of humanness leads to what Haslam calls “animalistic dehumanisation”: perceiving people as closer to animals, incapable of higher-level cognitive processes such as complex emotions or self-control. Infra-humanisation, as the process of denying outgroups human-specific emotions, is a major example of this dehumanisation process (Leyens et al., 2000).

Most social psychology studies on the topic have focused on an intergroup relations and discrimination perspective and mainly conceptualised dehumanisation as a strategy or justification for treating outgroup members badly. Researchers have been interested in what dehumanisation is (Haslam, 2006), the forms in which it manifests itself (Kteily and Landry, 2022), the effects it produces (with a strong focus on prejudice and a worst treatment of others more generally, see e.g., Bandura et al., 1975; for a review, see Haslam and Loughnan, 2016), and finally why it produces such effects. They have looked at explanations such as psychologically placing the outgroup outside the sphere of moral rules (Opotow, 1990), producing a sense of prejudice legitimisation when the outgroup is animalised (Bar-Tal, 2000), and morally disengaging from its suffering (Bandura et al., 1996; Bandura, 2002). Recently, other work has gone beyond this intergroup conflict perspective to encompass other views, notably in interpersonal relationships (see e.g., Haslam, 2022).

Surprisingly, however, to date dehumanisation has not been approached in social psychology as an *influence strategy*. As summarised above, research has focused on dehumanisation as a *process* leading the ingroup to mistreat outgroups, but never as a *strategy* to convince the ingroup to mistreat such an outgroup. This gap in research is extremely surprising given the recurrence throughout history of political propaganda using blatant animalised depictions of outgroups. From the colonisation of Aboriginal, native American, or African lands before the 20th century, to the direct extermination of the Jews before and during the Second World War, examples of animalistic dehumanisation of those who were displaced or eliminated are manifold (for a historical overview, see Smith, 2011). In all cases, animalised dehumanisation of the outgroup was used by political leaders to galvanise their own people and convince them that colonisation and/or extermination of other humans was legitimised, given the outgroup’s inferiority stemming from their animality. Similar depictions are still used today. For example, Israelis and Palestinians often and reciprocally depict each other as subhuman apes (Bruneau and Kteily, 2017).

In the present article, we investigate the potential influence effects that could stem from the animalistic dehumanisation of an outgroup. In social influence terms, we propose that depicting an outgroup in animalised terms could be an influence strategy leading to lasting unfavourable outgroup attitudes because it “immunises” ingroup members against subsequent counterinfluence attempts (such as pro-outgroup information or advocacy). In the following sections, we briefly discuss two key properties of animalistic dehumanisation that suggest this potential influence – namely, *essentialism* and *justification* – before turning to a theory of resistance to influence building on groups characteristics, that is, *psychologization* (Papastamou, 1986).

### 1.2. Essentialism and justification

With infra-humanisation theory, Leyens et al. (2000) posit that when people distinguish between their ingroup and an outgroup, they do not merely differentiate between the groups but also attribute them different essences. As a philosophical notion, *essentialism* refers to the belief that things have an essence, that is, certain necessary properties without which they could not be the things they are (Medin, 1989; Medin and Ortony, 1989). Essentialism is sometimes considered an adaptative way to give meaning to the world around us (Rhodes and Mandalaywala, 2017) and its use is not restricted to physical forms or categories but is also extended to non-physical contents or entities (Newman and Knobe, 2019).

By extension, psychological essentialism is the application of essentialism to humans, considering that humans represent some social categories to which they belong and therefore have some underlying properties that they share with other members – properties that are considered as causally responsible for their attributes (see Neufeld, 2022). At the intergroup level, this translates into the notion that groups have permanent and immutable properties, which determine their intrinsic and ontological nature (Yzerbyt et al., 1997), and therefore define what its members are – different groups potentially having different essences. Leyens and colleagues suggest that, to the extent that people do attribute such an essence to social groups, they would attribute a human essence to high-status groups, notably the ingroup, but an infrahuman (or animal) essence to inferior (out-)groups. The assumption is supported by research showing that people attribute more human emotions (or “secondary emotions,” referring mostly to sentiment) to ingroups than to outgroups, whom they deprive from this human essence (e.g., Leyens et al., 2001, 2003; Cortes et al., 2005). In recent years, research has expanded to study infra-humanisation with respect to a variety of groups and characteristics, including age (Boudjemadi et al., 2017), nationality (Davies et al., 2018), or religion (Banton et al., 2020), finding it to apply to a large set of possible categories (but see Enock et al., 2021).

Strictly speaking, these results on the attribution of human emotions show mainly a dehumanisation of the outgroup (who is denied cognitively advanced emotional processes) but no animalisation (which would have manifested itself in a greater attribution of primary or infrahuman emotions). Yet, one could argue that once an outgroup has been stripped from its human characteristics, what remains is the more basic characteristics shared with animals, that is, an animal essence. Essentialisation in this context is an essentialisation by proxy that relies on the characteristics that remain. Thus, a direct animalisation (i.e., depicting the outgroup in explicit animalised terms) should produce an even stronger animalistic essentialisation as it explicitly confers animal characteristics to the outgroup (as opposed to merely depriving them of human ones).

Moreover, it appears that animalistic dehumanisation is mainly associated to low-status, rather than high-status groups (Sainz et al., 2019a; see also Harris and Fiske, 2006, 2011). The fact that it mainly targets already inferior groups suggests that animalistic dehumanisation might be viewed as an easy and efficient strategy to explain such inferiority, justifying and strengthening a worse view of these groups as well as prejudice towards them, for example denying them equal access to resources through redistribution (Sainz et al., 2019b; Markowitz and Slovic, 2020; Sainz et al., 2020). The potential impact of animalistic dehumanisation as a justification tool is further supported by research showing that whilst having to justify intergroup relationships leads to an increased use of stereotypical traits (Hoffman and Hurst, 1990), the act of attributing such traits also facilitates this justification. Indeed, people readily use any information they believe they have about an outgroup or its individual members to justify prejudice against them (Yzerbyt et al., 1994).

What does this imply for intergroup attitudes and perception? Outgroups are very often depicted in negative terms compared to the ingroup, because of social identity needs (Tajfel and Turner, 1979) and the positive impact on self-esteem of a favourable relative difference between the groups (Hogg and Abrams, 1990; Rubin and Hewstone, 1998). However, the positivity and negativity attached to a group can change through time as intergroup relationships evolve and normative expectations change. In consequence, negative information about the outgroup can easily enough be compensated for by positive one. In contrast, essentialisation invokes permanent and immutable characteristics that define the group’s nature and thus cannot be changed (or at least are very hard to challenge subsequently). On this basis, we suggest that animalising an outgroup would constitute a very strong and lasting justification for mistreatment. Whilst positive information can compensate negative information, it cannot so easily erase the intrinsic animalistic properties of the group acquired through the animalisation process.

In terms of social influence, we therefore propose that animalistic dehumanisation as a propagandistic outgroup depiction strategy could lead to the attribution of durable negative traits that will stick to the outgroup like a lasting stench, making influence targets resistant to counterpropaganda attempts in favour of the outgroup. Put simply, animalistic dehumanisation could be a potent influence strategy as a propaganda tool against an outgroup, with one core property: because of the immutable nature of the characteristics it attributes to the outgroup, it would immunise against subsequent opposed persuasive messages highlighting positive characteristics of the outgroup – something a classical mere negative influence message would not do. Contrary to a merely negative message, a subsequent positive message would not be enough to counter the animal characteristics attached to the group as an essence, which would become a permanent justification for treating the animalised group badly and thus for derogating any message that would argue the opposite.

### 1.3. Psychologization as an attributional theory of resisting influence

Our proposition relies on an attributional strategy whose purpose is to resist influence. Different theories have developed around the notion of resisting influence (e.g., McGuire, 1964; Chen et al., 1992). Crucially for the present purpose, one such theory directly and explicitly relies on a similar form of attributional strategy as the one we propose here, namely: psychologization theory (Papastamou, 1986; Papastamou and Mugny, 1987). Our proposition, however, is somewhat different. In the following section we describe and summarise psychologization theory before showing what distinguishes it from the present proposition.

Psychologization theory postulates that social groups can use an attributional strategy to ruin the credibility of a source of influence deemed undesirable, and therefore protect their members from such influence. Called “naturalisation” at a general level (Papastamou et al., 1980), this strategy aims to explain – or attribute – opinion divergence between ingroup members and an undesirable source of influence (conceptualised in the theory as the majority and as a minority, respectively) through some intrinsic properties of the latter.

Of the several forms this process can take, *psychologization* consists in establishing a link between the ideological position defended by the source of influence and psychological characteristics that are specific to that source, whilst suggesting that the latter explain the former. In other words, the source’s characteristics become the reason behind its message. Psychologization thus distorts the perception of the source and of its discourse. By attributing its position to some idiosyncratic specificity, it denies the possibility that the discourse might convey an alternative or emergent reflection of reality that deserves attention. Instead, it implies that the discourse can be simply ignored. To give a concrete example, if a source were advocating for a strong control of industrial emissions to reduce pollution, psychologization would consist in attributing its position to some innate authoritarian trait, making it sound as if the source wanted to control the industry rather than to protect the environment (Papastamou et al., 1992). Accordingly, research has shown that psychologization considerably reduces a source’s potential for influence (e.g., Mugny and Papastamou, 1980; Papastamou et al., 1980; Mugny et al., 1983).

Psychologization and animalisation share similarities: both rely on attributional processes and more specifically on the attribution of some idiosyncratic characteristics to an undesirable (out)group. There are, however, two main differences in an intergroup context showing that our proposition cannot be subsumed to mere psychologization. First, psychologization is meant as a strategy to derogate the source of a persuasive message. It can only arise if and when a source tries to influence ingroup members and make them change their opinions about the outgroup. Animalisation, in contrast, is meant to justify discrimination against an outgroup regardless of whether this group was expressing any diverging opinion. Put differently, animalisation utilises intrinsic animalistic characteristics to explain why the outgroup *is* what it is, whilst psychologization utilises idiosyncratic psychological specificities to explain why the source *says* what it says. Second, psychologization specifically targets a source that advocates in favour of its own (minority or stigmatised) group. It was not theorised to apply to a third-party source that would argue in favour of the stigmatised outgroup. In other words, psychologization would only be effective in an intergroup context (in the sense of blocking the source’s influence) if the source belongs to the outgroup it is advocating for. This gives an “advantage,” as far as efficacy is concerned, to animalisation strategies. Indeed, animalisation can occur regardless of the relationship between the source and the outgroup it is defending. It could theoretically block subsequent counterpropaganda attempts just as well if those arise from a source belonging to the outgroup or one that is external to the outgroup.

In summary, we propose that animalistic dehumanisation functions as an attributional strategy (like psychologization) leading to a lasting negative perception of an outgroup and justifying the expression of prejudice against it. In addition, and precisely because of the permanent animal essence it confers to the outgroup, animalistic dehumanisation should last over time and block subsequent counterpropaganda attempts.

### 1.4. The present study

We present here the results of an experimental study that tests this contention. The study included three experimental conditions: negative animalisation vs. negative humanisation vs. positive humanisation. We aimed to investigate the specific effect of animalisation, distinguishing it from a mere negative (but still humanised) depiction of the group. With respect to the literature review above, we expected that animalisation would convey longer-lasting negative connotations linked to the essence of the group, therefore leading to prejudice that would resist a subsequent counterpropaganda attempt. The negative depiction, however, would be more easily compensated for by the presentation of new positive information. Therefore, we hypothesised a unique effect of animalisation that would be different from both positive and negative humanised depictions.

The study was conducted in 2011 in Switzerland and we focused on a minority group that was particularly relevant at this time and place: Roma beggars. Roma beggars were then considered as a very low status group in society compared to national citizens. They were also considered a disruptive group, not integrating well in the country. We therefore created a first “propaganda” message that depicted Roma beggars in negative animalised terms, or in negative or positive humanised terms (see detail of the manipulation below), and a second “counterpropaganda” message that defended Roma beggars’ rights.

Given the potential disruptive nature of this outgroup, we suspected that men would be more inclined to discriminate against Roma beggars than women. This was derived, first, from the “male warrior hypothesis” suggesting that men respond more strongly to outgroup threats directed towards the ingroup or its norms (e.g., McDonald et al., 2012) and, second, from findings showing that men have a stronger social dominance orientation than women (e.g., Pratto et al., 2000; Sidanius et al., 2000; Levin, 2004) – the two effects probably being linked (Sugiura et al., 2017). It appears that individuals higher on SDO tend to animalise outgroups to a greater extent (see Haslam and Loughnan, 2014). In addition, as dominant groups might be more inclined to express prejudice (Jost and Banaji, 1994), they would easily answer to a threat by expressing more prejudice. We therefore expected men could react more strongly to an animalisation manipulation. Congruently with this reasoning, some findings suggest that men who animalise women to a greater extent are also more inclined to adopt negative behaviours against women, whilst women do not show such variation (Rudman and Mescher, 2012). Foreseeing that the procedure would make it difficult to recruit a very large number of participants (see below), we anticipated that the sample size might not yield enough statistical power to formally test for a moderating effect of sex. We therefore decided to recruit only male participants as they were the one expected to react more strongly towards outgroup members after their animalisation.

Finally, it should be noted that as this study was conducted in 2011, it conformed to the research standards of that time. Accordingly, and unfortunately, sample size was determined based on rule of thumb rather than on an *a priori* power analysis. The sample size might also look relatively low compared to today’s best practises. We come back to these potential limitations in the discussion. At the time, the authors’ university did not have an institutional review board nor was any formal ethics approval required. Nevertheless, the research respected the principles put forward in the Declaration of Helsinki. Participation was voluntary with the option to opt out at any time, all data collected were confidential and anonymous, participants gave their informed consent before starting, and were fully debriefed at the end of the study. Finally, potential for harm was minimal.

## 2. Method

### 2.1. Participants and procedure

Participants were passers-by approached in the street in a medium-sized city who agreed to fill a pencil-and-paper questionnaire studying “people’s impressions of Roma beggars” (completion took around 10 min). Participation was on a voluntary basis with no monetary compensation. As described above, we aimed to recruit only male participants. Only male-looking passers-by were approached. Participants’ gender was further verified in the questionnaire and all participants did indeed self-describe as men. The sample included 81 men of a mean age of 27.47 years (*SD* = 7.78). A sensitivity power analysis indicated that this *N* allowed to detect a medium-size effect (*η*_p_^2^ = 0.10) with 80% power (analysis run on GPower 3.1.9.7, calibrated for the test of the linear contrast of the hypothesis, one-tailed).

The questionnaire was structured as follows: participants first read the *propaganda message* (anti-Roma beggars), which served as the experimental manipulation. Participants were randomly allocated to one of three versions of the text in a between-subject design (negative animalisation, *n* = 25 vs. negative humanisation, *n* = 26 vs. positive humanisation, *n* = 30). Following the text they answered manipulation checks. They then moved on to the second text, the *counterpropaganda message* (favourable to Roma beggars), also followed by manipulation checks. They finally completed a set of scales measuring their attitudes and behavioural intentions towards Roma beggars (see detail below). Unless stated otherwise, all items used a 7-point Likert scale (1 = *Not at all*, 7 = *Absolutely*).

### 2.2. Material

#### 2.2.1. Experimental manipulation: Animalisation and humanisation

The experimental manipulation (3 conditions: negative animalisation vs. negative humanisation vs. positive humanisation) was introduced in the first text given to the participants. The text was presented as an (alleged) newspaper excerpt, more specifically as the first paragraph of a personal testimony published in the readers’ letters section of a local newspaper. Its author described how he had witnessed a group of Roma beggars gathering at the train station in the early morning, taking breakfast together and organising for the day. The structure of the text was the same across experimental conditions but key words were varied to present Roma beggars in a negative-animalised vs. negative-humanised vs. positive-humanised way, respectively (translated from the original study language to English):

A group of Roma beggars were organising themselves for the day. They were [*barking away / chatting away / chatting away*] and having breakfast. Their manner of eating was rather [*beast-like / unsophisticated / sober*]. Besides, when sharing the food each one was [*fighting to get the best share / stubbornly insisting on having the best share / open to give the best share to someone else*]. When interacting with each other, these Roma beggars showed [*no docility / no deference / deference*] to others, sign of a [*wild / independent / solidarity*] attitude. They also exhibited [*instinctive and impulsive / irrational and spontaneous / reasoned and thoughtful*] behaviour.

#### 2.2.2. First manipulation checks

Four items served as manipulation checks. Two items measured the valence of the image of Roma beggars conveyed in the text: “Upon reading this text, would you say that the image it gives of Roma beggars is… (1) Positive, (2) Negative.” After recoding the positive item, the two were aggregated into an average single score of *negative perception* (Pearson’s correlation: *r*(79) = −0.87, *p* < 0.001). Two other items measured the degree of animality-humanity conveyed by Roma beggars: “Upon reading this text, would you say that the image it gives of Roma beggars is … (1) Repugnant, (2) Disgusting^1^.” The two items were aggregated into an average score of *animalistic perception* (*r*(79) = 0.60, *p* < 0.001). For the sake of the cover story, these items were embedded into a larger list of questions about Roma beggars which served as filler (e.g., “friendly,” “warm”).

#### 2.2.3. Counterpropaganda information

Participants then read a second text, an alleged post written by a human rights lawyer, also published in a local newspaper. The text (386 words) consisted in several excerpts from the alleged longer post, selected to convey its core meaning.

In essence, the text started by exposing the issue, stating that the increasing presence of Roma beggars was arousing a legitimate debate but that the heavy-hand reaction of the authorities might go against their basic human rights. It went on to advocate Roma beggars’ human rights, reaffirming that they, just like any other human being, was entitled to the same treatment and opportunities as everyone else. The text then blamed the dire situation of Roma beggars on the discrimination of which they were victim, rather than the other way around, citing statistics that showed that Roma people fully integrated into society when they were not discriminated against. The text concluded on an appeal to confer Roma beggars the same opportunities as everyone else.

#### 2.2.4. Second manipulation check (check of persistence)

Participants were presented a second time with the same list of questions about Roma beggars, although this time the questions asked about their personal impressions rather than the image conveyed in the text (i.e., “What is your personal image of Roma beggars?”). The aim was to ensure that the negative-human framing effect would have been countered by the new, pro-Roma beggars message, but the animalisation framing—with respect to our hypothesis that animalisation immunises against counterpropaganda attempts—would not. Participants therefore answered again the two questions pertaining to negative perception [*r*(79) = −0.78, *p* < 0.001] and the two questions pertaining to animalistic perception [*r*(79) = 0.75, *p* < 0.001] – again aggregated into their respective average scores.

#### 2.2.5. Dependent variables

Several questions assessed participants’ attitudes towards Roma beggars through various angles. Drawing from hierarchy enhancing strategies that can be used to justify the Roma beggars’ inferiority, we first measured *acceptance of Roma beggars’ superior position in the workplace*. Imagining that a former Roma beggar would be hired at their workplace, participants reported how acceptable it would be for them that this person (a) was offered a job with a higher salary than themselves, (b) was offered a job with a lesser salary than themselves, (c) became their line manager, (d) that *they* became the line manager of this former Roma beggar. Questions (a) and (c) were aggregated into an index of acceptance of Roma beggars’ superior position in the workplace [*r*(79) = 0.78, *p* < 0.001]. Questions (b) and (d) were aggregated into an index of acceptance of Roma beggars’ inferior position in the workplace [*r*(79) = 0.63, *p* < 0.001]. These measures can be considered as relative social comparison choices. Indeed, the first one is similar to upward comparison (i.e., to a better-off other) that might be threatening (e.g., Tesser, 1988) and trigger low acceptation. The second one is similar to downward comparison (i.e., to a less well-off other), a comparison often used as a strategy to respond to threats (see Wills, 1981). We had no *a priori* hypothesis about the specific effect of the animalisation manipulation on this variable and considered it equally likely that animalisation would lead to a lower acceptance of others’ superior position, or to a greater acceptance of others’ lower position – or to both.

Second, we measured *discriminatory hiring intentions*. Participants indicated how likely they would be to hire a former Roma beggar for four different occupations, two lower status (receptionist and night watchman) and two higher status ones (intermediate executive and accountant). Following the same logic as above, we aggregated those in separate pairs of two indicating willingness to hire for low-status [*r*(79) = 0.55, *p* < 0.001] and high-status jobs [*r*(79) = 0.84, *p* < 0.001]. These measures had a similar purpose as the acceptance of superior/inferior position described just above, but with a focus on the status granted to Roma beggars through hiring. Again, we did not have *a priori* hypothesis as to whether animalisation would rather lead to a lower willingness to hire Roma beggars for higher status jobs, or to a higher willingness to hire them for lower status jobs, or both.

Thirdly, we measured *attitudes towards State interventions in favour of Roma beggars*. Participants indicated how much they would support State interventions aiming to help Roma beggars (a) get jobs, (b) get lodging, (c) exit poverty, and (d) they would support an active implication of the State to reduce discrimination against Roma beggars. The four questions were aggregated into a single average attitudinal index (Cronbach’s *α* = 0.82).

Finally, participants answered a *social proximity* measure. Three questions assessed their willingness to accept Roma beggars living (a) in their neighbourhood, (b) in their building, and (c) on the same floor as them. The three questions were aggregated into a single index of acceptance of social proximity (Cronbach’s *α* = 0.92).

## 3. Results

### 3.1. Analysis strategy

All analyses relied on linear regression models with the experimental manipulation entered as a set of Helmert contrasts. For the negative perception index (manipulation check), we used a contrast testing a *valence effect*, that is, opposing the positive humanisation condition (coded +2) to the two negative conditions, animalised (coded −1) and humanised (coded −1). The orthogonal contrast (0, −1, +1) was also entered in the analysis.

For all other dependent measures, including the animalistic perception index, the contrast tested an *animalisation effect*, opposing the negative animalisation condition (coded −2) to the two humanisation conditions, negative (coded +1) and positive (coded +1). Again, the orthogonal contrast (0, −1, +1) was entered in all analyses.

When two measures pertain to a similar construct (e.g., acceptance of Roma beggars’ superiority vs. inferiority in the workplace, or hiring intentions for a high vs. low status job), we computed a difference score between the two measures to first explore whether the effect of the manipulation was similar or different on the two measures (let us note that the main effect of the manipulation obtained in a linear regression on such a difference score is strictly equivalent to the interaction term [manipulation × measure] in a repeated-measure analysis of variance), before turning to separate analyses on each measure.

### 3.2. Manipulation checks

#### 3.2.1. Negative perception index

To test how the first message (negative animalisation vs. negative humanisation vs. positive humanisation) influenced negative perception, directly after reading the text as well as after having read the second counterpropaganda message, we computed a difference score of the two negative perception indices (post – pre). A regression analysis using the *valence effect* set of contrasts described above revealed a significant effect of the key contrast, *b* = −0.76, 95% CI [−1.02, −0.49], *t*(76) = −5.84, *p* < 0.001, *η*_p_^2^ = 0.31, suggesting a different evolution of negative perceptions between conditions. The orthogonal contrast was not significant, *b* = 0.12, 95% CI [−0.35, 0.58], *t*(76) = 0.51, *p* = 0.61, *η*_p_^2^ < 0.001.

We then turned to separate analyses for each of the negative perception indices (see Table 1). On the first measure, taken directly after the initial message, the *valence effect* contrast again proved significant, *b* = −0.70, 95% CI [−0.90, −0.49], *t*(77) = −6.62, *p* < 0.001, *η*_p_^2^ = 0.36 (the orthogonal contrast was not significant, *b* = −0.07, 95% CI [−0.44, 0.32], *t*(77) = −0.36, *p* = 0.72, *η*_p_^2^ = 0.00). In other words, the two negative conditions (animalised and humanised) produced a more negative perception of Roma beggars than the positive humanisation condition.

**Table 1:** Mean scores (and standard deviations) of negative perceptions (higher scores indicate more negative perceptions) and animalistic perceptions measured directly after the initial propaganda message and after the counterpropaganda message.

	Negative animalisation	Negative humanisation	Positive humanisation
**Manipulation checks**	**M (SD)**	**M (SD)**	**M (SD)**
*Negative perception index*			
After the initial propaganda	5.56 (1.36)	5.42 (1.02)	3.40 (1.61)
After the counterpropaganda	4.70 (1.59)	4.32 (1.26)	4.81 (1.42)
*Animalistic perception index*			
After the initial propaganda	4.78 (2.43)	3.62 (1.54)	2.00 (1.30)
After the counterpropaganda	3.28 (1.66)	2.60 (1.26)	2.57 (1.57)

On the second measure taken after exposure to the counterpropaganda message, however, no significant effect emerged, respectively for the key contrast: *b* = 0.10, 95% CI [−0.12, 0.32], *t*(77) = 0.89, *p* = 0.37, *η*_p_^2^ = 0.01, and for the orthogonal contrast: *b* = −0.19, 95% CI [−0.58, 0.22], *t*(77) = −0.93, *p* = 0.35, *η*_p_^2^ = 0.01. This suggests that the counterpropaganda message cancelled the initial negative effect produced by the experimental manipulation.

Moreover, the negative animalisation condition showed a significant decrease between the two times of measure, *b* = 0.86, 95% CI [0.10, 1.63], *t*(77) = 2.25, *p* = 0.028, as did the negative humanisation condition, *b* = 1.10, 95% CI [0.36, 1.83], *t*(77) = 3.00, *p* = 0.004. The positive humanisation condition, on the other hand, revealed a difference going on the opposite direction, *b* = −1.28, 95% CI [−1.90, −0.67], *t*(77) = −4.14, *p* < 0.001.

#### 3.2.2. Animalistic perception index

A similar analysis was run on animalistic perception indices, using first a difference score between the two indices (post – pre). The *animalisation effect* contrast yielded a significant effect on the difference score, *b* = −0.45, 95% CI [−0.76, −0.14], *t*(76) = −2.87, *p* = 0.005, *η*_p_^2^ = 0.10, as did the orthogonal contrast, *b* = −0.87, 95% CI [−1.39, −0.34], *t*(76) = −3.28, *p* = 0.002, *η*_p_^2^ = 0.12.

We then turned to separate analyses for each index (see Table 1). On the first measure, taken directly after the persuasive message, the *animalisation effect* contrast was again significant, *b* = −0.66, 95% CI [−0.94, −0.37], *t*(77) = −4.55, *p* < 0.001, *η*_p_^2^ = 0.21, and so was the orthogonal contrast, *b* = −0.81, 95% CI [−1.29, −0.33], *t*(77) = −3.33, *p* = 0.001, *η*_p_^2^ = 0.13. This indicates that the manipulation of animalisation and of negativity produced cumulative effects, resulting in the highest animalistic perception in the negative animalisation condition, followed by the negative humanisation condition, and finally the positive humanisation condition.

Crucially, on the second measure taken after exposure to the counterpropaganda message, only the animalisation effect contrast remained – and only marginally – significant, *b* = −0.23, 95% CI [−0.47, 0.01], *t*(77) = −1.92, *p* = 0.059, *η*_p_^2^ = 0.05, whilst the orthogonal contrast effect disappeared, *b* = −0.01, 95% CI [−0.42, 0.40], *t*(77) = −0.07, *p* = 0.95, *η*_p_^2^ = 0.00. In other words, whilst the negative animalisation presentation produced a more animalistic perception of Roma beggars that somewhat resisted the counterpropaganda message, animalistic perception in the negative humanisation condition disappeared after the counterpropaganda, reverting to levels similar to those of the positive humanisation condition.

Moreover, the negative animalisation condition showed a significant decrease between the two times of measure, *b* = 1.50, 95% CI [0.67, 2.32], *t*(77) = 3.64, *p* < 0.001, as did the negative humanisation condition, *b* = 1.02, 95% CI [0.18, 1.85], *t*(77) = 2.43, *p* = 0.017. The difference in the positive humanisation condition went in the opposite direction but was only marginally significant, *b* = −0.71, 95% CI [−1.44, 0.02], *t*(77) = −1.95, *p* = 0.055.

### 3.3. Dependent measures

#### 3.3.1. Attitudes relative to hierarchy and salary in the workplace

Following the same analysis strategy, we then continued with analyses on the main dependent measures, turning first to attitudes in the workplace. A visual depiction of the results is available in Electronic Supplementary Figures 1–6. To better distinguish acceptance of superior versus inferior positions in the workplace, we first considered a difference score between the two measures (superior – inferior positions). The contrast testing an animalisation effect yielded a significant effect, *b* = 0.35, 95% CI [0.08, 0.63], *t*(78) = 2.62, *p* = 0.011, *η*_p_^2^ = 0.08, whilst the orthogonal contrast did not, *b* = 0.04, 95% CI [−0.41, 0.50], *t*(78) = 0.19, *p* = 0.85, *η*_p_^2^ = 0.00, suggesting a difference between the two measures depending on the experimental conditions.

We then turned to separate analyses for each index (see Table 2). Looking at acceptance of Roma beggars’ superiority in the workplace, first, the animalisation effect contrast was significant, *b* = 0.39, 95% CI [0.11, 0.66], *t*(78) = 2.81, *p* = 0.006, *η*_p_^2^ = 0.09, whilst the orthogonal contrast was not, *b* = 0.15, 95% CI [−0.31, 0.61], *t*(78) = 0.65, *p* = 0.52, *η*_p_^2^ = 0.005. This indicates that participants in the negative animalisation condition expressed a lower acceptance of having a Roma beggar occupying a position superior to their own than participants in the other two conditions, who did not differ from one another.

**Table 2:** Effect of the experimental manipulation on the different dependent measures: acceptance of Roma beggars’ superior and inferior positions in the workplace, hiring intentions for high and low-status jobs, attitudes towards State interventions, and acceptance of social proximity.

	Negative animalisation	Negative humanisation	Positive humanisation
**Dependent measures**	**M (SD)**	**M (SD)**	**M (SD)**
*Acceptance of Roma beggars’ positions in the workplace*			
Roma beggars in superior positions	3.62 (1.52)	4.63 (1.95)	4.93 (1.67)
Roma beggars in inferior positions	4.44 (0.97)	4.42 (1.02)	4.63 (1.47)
*Hiring intentions*			
Hiring intentions for a high-status job	3.80 (1.93)	4.71 (1.83)	4.98 (1.75)
Hiring intentions for a low-status job	4.46 (1.59)	4.87 (1.67)	5.35 (1.42)
*Attitudes towards State interventions*	5.12 (1.23)	5.40 (1.20)	5.27 (1.45)
*Acceptance of social proximity*	3.55 (1.55)	4.00 (1.38)	4.61 (1.57)

On acceptance of Roma beggars’ inferiority in the workplace, however, none of the effect reached significance (respectively, animalisation effect contrast: *b* = 0.03, 95% CI [−0.17, 0.22], *t*(78) = 0.31, *p* = 0.76, *η*_p_^2^ = 001; orthogonal contrast: *b* = 0.11, 95% CI [−0.21, 0.43], *t*(78) = 0.66, *p* = 0.51, *η*_p_^2^ = 0.006). Thus, acceptance of having a Roma beggar occupying a position inferior to one’s own was not impacted by the manipulation.

#### 3.3.2. Discriminatory hiring intentions

As before, we computed a difference score between intentions to hire a Roma beggar for a low-status versus high-status position (high – low). On this difference score, none of the effects was significant (animalisation effect contrast: *b* = 0.13, 95% CI [−0.11, 0.38], *t*(78) = 1.08, *p* = 0.28, *η*_p_^2^ = 0.02; orthogonal contrast: *b* = −0.11, 95% CI [−0.52, 0.30], *t*(78) = −0.52, *p* = 0.61, *η*_p_^2^ = 0.003), indicating that any effect of the experimental conditions, if any, would be similar on the two measures (Table 2).

Turning to hiring intentions for a high-status job, specifically, the animalisation effect contrast proved significant, *b* = 0.35, 95% CI [0.05, 0.64], *t*(78) = 2.37, *p* = 0.020, *η*_p_^2^ = 0.07, whilst the orthogonal contrast did not, *b* = 0.14, 95% CI [−0.36, 0.63], *t*(78) = 0.55, *p* = 0.58, *η*_p_^2^ = 0.004. This indicated that participants in the negative animalisation condition were less willing to hire Roma beggars for a high-status job than participants in the other two conditions, who did not differ from one another.

Explaining the absence of statistical difference between the two measures, a similar result appeared on hiring intentions for a low-status job, although the effect was weaker and only marginally significant, *b* = 0.22, 95% CI [−0.04, 0.47], *t*(78) = 1.73, *p* = 0.088, *η*_p_^2^ = 0.04 (orthogonal contrast: *b* = 0.24, 95% CI [−0.18, 0.65], *t*(78) = 1.16, *p* = 0.25, *η*_p_^2^ = 0.02).

#### 3.3.3. Attitudes towards state interventions in favour of Roma beggars

Analyses on attitudes towards State interventions yielded no significant results (animalisation effect contrast: *b* = 0.07, 95% CI [−0.14, 0.28], *t*(78) = 0.68, *p* = 0.50, *η*_p_^2^ = 0.006; orthogonal contrast: *b* = −0.07, 95% CI [−0.42, 0.29], *t*(78) = −0.38, *p* = 0.71, *η*_p_^2^ = 0.002), suggesting that the experimental manipulation did not impact these attitudes (see Table 2).

#### 3.3.4. Social proximity

The final dependent measure we considered was acceptance of social proximity. On this measure, the analysis showed a significant effect of the animalisation contrast, *b* = 0.25, 95% CI [0.01, 0.49], *t*(78) = 2.10, *p* = 0.039, *η*_p_^2^ = 0.05, but not of the orthogonal contrast, *b* = 0.31, 95% CI [−0.10, 0.71], *t*(78) = 1.52, *p* = 0.13, *η*_p_^2^ = 0.03. Accordingly, participants in negative animalisation condition were less inclined to accept social proximity with Roma beggars than participants in the other two conditions, who did not differ from one another (Table 2).

## 4. General discussion

### 4.1. The present results

In the present research we tested the effect of an animalistic depiction of a disliked outgroup – Roma beggars – as compared to negative (and positive) but humanised depiction. Drawing from theories of dehumanisation, we hypothesised that an animalistic depiction would have a unique, longer-lasting effect against counterpropaganda than a mere negative depiction. We tested this effect, first, on participants’ perception of the outgroup in more or less negative, and more or less animalistic terms, before turning to their attitudes and behavioural intentions towards the outgroup.

Without surprise, the two negative messages (animalising and humanising) were perceived as conveying an equally negative image of the outgroup. Feelings of disgust and repugnancy elicited by the messages showed a cumulative effect of negativity and animalisation: scores were lowest in the positive humanised condition, intermediate in the negative humanised condition, and highest in the negative animalised condition. Interestingly, only the animalised condition maintained its effect in the longer run. Specifically, and strikingly, the animalised message conferred resistance against the subsequent positive counterpropaganda and translated into an enduring personal perception of the group as more disgusting and repugnant. The effect of the negative but humanised depiction, in contrast, was cancelled out by the positive propaganda and results in as little disgust/repugnancy as the positive humanised depiction. In contrast, the negative personal perceptions did not resist the counterpropaganda, regardless of the experimental condition: all participants reported more positive personal perceptions after the counterpropaganda message. These results show that the negative animalisation message leads to a “sticking,” lasting perception that expresses itself in animalistic rather than simply negative terms. In sum, animalisation seems to activate more durable characteristics (probably related to a permanent essence attributed to the group), which opens the possibility of a protective effect against counterpropaganda.

An odd and unexpected result seems to appear with the negative perception index in the positive humanisation condition specifically: negative perceptions tend to *increase* following the presentation of the second, positive message. This suggests that the accumulation of two positive influence messages (the first depicting the stigmatised outgroup in a positive and human way, and the second advocating for their human rights) elicited some reactance amongst our participants (Brehm, 1966), who might perceive the process as too-forced an attempt to influence them.

Above and beyond the effect on negative perception, the animalised message led in turn to more negative attitudes and a greater willingness to discriminate against Roma beggars, something the negative but humanised depiction did not trigger (this condition being not different statistically from the positive humanised condition).

This effect appears on three out of four measures. It was found nonsignificant for the measure of attitudes towards State interventions in favour of Roma beggars (although the means, descriptively, go in the expected direction). The nonsignificant results on this measure are rather peculiar given that the literature indicates that the animalisation of poor people leads to a reduced inclination towards wealth redistribution through State interventions (see Sainz et al., 2019b). As such, we do not have a clear explanation for the lack of effect on this specific measure, except the relatively low statistical power of the study.

Moreover, comparative results on attitudes showed an interesting pattern. Specifically, participants made a clear distinction between superior and inferior positions in the workplace. Participants exposed to the negative animalised depiction were reluctant to see Roma beggars occupy higher status positions or earning higher salary than themselves. However, they were similarly in favour of the idea of them occupying lower status positions / earning lower salary than participants in the other conditions. This result indirectly speaks to similar findings in the intergroup literature: it is congruent with work showing an asymmetry in ingroup favouritism as people produce higher ingroup favouritism when it comes to allocating “positive resources” but are reluctant to treat groups differently when it comes to “negative resources” (e.g., Mummendey et al., 1992) – whatever the precise underlying mechanisms might be (Brewer, 1999; Otten and Mummendey, 1999). This result is also congruent with the infra-humanisation perspective (Leyens et al., 2000), which shows that people specifically reduce the human emotions attributed to outgroups relative to the ingroup (i.e., denying them what allows to reach human superiority) but do not attribute them more animal emotions (i.e., giving them traits that confine them to inferiority). The common pattern here might suggest a possible similarity in underlying motives and processes.

### 4.2. Limitations and future directions

Some limitations to the present work must be acknowledged. First, we focused here on animalistic dehumanisation given its prominence in human history as a propaganda strategy to justify the bad treatment of outgroups. However, as we noted in the introduction, there are two forms of dehumanisation, namely the animalistic and the mechanistic one (Haslam, 2006). The present results are limited to animalistic dehumanisation, and whether or not a depiction in negative mechanistic terms would produce similar effects remains an open question.

Second, and as noted previously, the sample size of the present study was somewhat limited, yielding power to detect only medium-size effects. The consistency in the results across dependent variables seems to indicate that power was sufficient to address the current research question. However, the design might have been underpowered when it comes to contrasting the negative animalisation and the negative humanisation condition. Interpretation of the difference between these conditions needs caution and we cannot rule out that the effect is in fact a linear one, the negative humanised condition leading (after exposure to a positive counterpropaganda) to slightly more negative attitudes than the positive humanised condition. Future research is needed to explore these distinctions further.

Third, the sample is composed of men only. We do not know whether women would produce the same pattern and thus whether the effect can be generalised. As stated in the Methods section, we suspect that women might be less sensitive to animalistic depictions – at least when it comes to attitude- or prejudice-grounded content. Future research will need to include female participants to verify whether they react the same or differently as men, and additionally investigate the effect of participants’ gender on other, non-prejudice-grounded, contents.

Fourth, the study did not include a control group without any initial message. This might have been necessary to more clearly understand whether the difference across conditions is due to an increase of prejudiced intentions in the negative animalisation condition, or a decrease of it in the positive humanisation condition, or both. Yet, we would argue that the negative humanisation condition serves the role of this “control” condition. Given that this condition creates a more negative perception than the positive humanisation condition prior to the counterpropaganda message *but* similar perceptions and attitudes after the counterpropaganda, it seems that the positive humanisation condition does not have any specific positive effect on attitudes – rather, it is merely just as positive as the combination of an initial negative depiction counterbalanced by a positive counterpropaganda. We would therefore argue that the effects seem to be driven by the animalisation condition instead.

Last but not least, and relatedly, we must note again that the data presented here is now more than 10 years old (collected in 2011). With the constant evolution of social norms regarding the public expression of prejudice, it is unclear how these results would replicate today for a similar outgroup. First, if the animalisation of stigmatised minorities might have been acceptable a decade ago, it is becoming less and less so nowadays. We suspect that as of today, a blatant animalisation strategy might not work and could even produce a backlash effect against those who used it as an influence strategy. Second, the results might be constrained to those few minority groups that are *not* protected by stronger equalitarian and human rights. The current climate is one of attune sensibility to groups who, just like Roma beggars (Caflisch, 2017), have suffer from stigmatisation and discrimination until recently – or still do. Future research will need to test how replicable the results are as of today, and for which groups exactly.

### 4.3. Extensions and conclusion

The last limitation mentioned above suggests that dehumanisation as a protection strategy against counterpropaganda might be more effective for minority groups that are *not* protected in the Zeitgeist. In this respect, high-status minorities such as the “1%,” Wall Street bankers, politicians and so on, could form a specific target for which dehumanisation would lead to lasting derogating perceptions that resist counterinfluence. However, research shows that such high-status groups are more subjected to mechanistic than animalistic dehumanisation (Sainz et al., 2019a). Accordingly, it is possible that animalistic dehumanisation as an influence-blocking strategy might not be so efficient with respect to these groups – but mechanistic dehumanisation would be. This is also congruent with findings that high-status groups (mainly rich groups) are considered as low on warmth but high on competence (Durante et al., 2017), which corresponds to traits that might be attributed to machines. In sum, above and beyond a mere replication of the present findings, future research should investigate whether the results hold with different dehumanisation strategies (animalistic vs. mechanistic) and for which target group. Based on the current literature we tentatively hypothesise that animalistic dehumanisation would confer a better protection against counterinfluence for low-status groups, whilst mechanistic dehumanisation would do so for high-status groups.

Moreover, whilst a blatant animalisation strategy might backfire nowadays, we suspect that more subtle activations of animalisation might still prove effective given that people probably strongly associate animality to outgroups by default. Indeed, Leyens et al. (2000) consider that essentialisation – which is at the core of animalistic dehumanisation – is a key feature of categorisation. They argue that categorising someone in an outgroup necessarily results in denying them humanity to an extent. Therefore, it is possible that merely making salient the association between animality and an outgroup, even in a subtle way, might confer protection against counterpropaganda. In other words, in the same way that people share and automatically activate stereotypes about outgroups (e.g., Devine, 1989), subtle activations of animality might still immunise against counterinfluence even in the absence of a direct animalised depiction of the target outgroup. If that were indeed the case, it would make dehumanisation a very powerful tool against social influence from opposite views – something political actors have clearly assumed for a very long time.

In conclusion, and at a more general level, the present work opens new avenues for research on dehumanisation, to study it as an influence process rather than solely as an intergroup phenomenon. It is essential to start filling the gaps to understand this protective property of animalistic dehumanisation. Indeed, those utilising such strategies could get a strong advantage in the long run, as the results suggest that they are difficult to counterinfluence, which can lead to dire consequences for its victims. In a second step, research also needs to move to identifying and understanding ways to cancel the effects of animalisation, and to provide those who oppose it with a reciprocal tool to successfully counter its nefarious and harmful effects.

## Data availability statement

The raw data supporting the conclusions of this article will be made available by the authors, without undue reservation.

## Ethics statement

At the time this study was conducted, ethical review and approval was not required for the study on human participants in accordance with the local legislation and institutional requirements. The patients/participants provided their written informed consent to participate in this study.

## Author contributions

AQ: conceptualization, methodology, investigation, formal analysis, writing—original draft, and writing—review and editing. FL: formal analysis, writing—original draft, and writing—review and editing. All authors contributed to the article and approved the submitted version.

## Funding

Open access funding was provided by the University of Geneva.

## Conflict of interest

The authors declare that the research was conducted in the absence of any commercial or financial relationships that could be construed as a potential conflict of interest.

## Publisher’s note

All claims expressed in this article are solely those of the authors and do not necessarily represent those of their affiliated organizations, or those of the publisher, the editors and the reviewers. Any product that may be evaluated in this article, or claim that may be made by its manufacturer, is not guaranteed or endorsed by the publisher.

## Footnotes

^1^Animals, especially vermin or dangerous species, have been shown to elicit such feelings (e.g., Davey and Marzillier, 2009), which are associated with fearing them (Polák et al., 2020).
