# Erratum: Animalistic dehumanisation as a social influence strategy

**DOI:** 10.3389/fpsyg.2023.1170205

**Published:** 2023-03-03

**Authors:** Frontiers Production Office

**Affiliations:** Frontiers Media SA, Lausanne, Switzerland

**Keywords:** dehumanisation, animalisation, influence, resistance, essentialism, justification

An omission to the funding section of the original article was made in error. The following sentence has been added: “Open access funding was provided by the University of Geneva.”

The publisher apologizes for this mistake. The original article has been updated.

