# Supplementary material for: Animalistic dehumanisation as a social influence strategy
Source: Front Psychol. 2023 Jan 11;13:999959. doi: 10.3389/fpsyg.2022.999959 (PMC9875809; doi:10.3389/fpsyg.2022.999959)
Supplement: Supplementary file 1 [file Data_Sheet_1.PDF]

Supplementary Material

1 Supplementary Figures

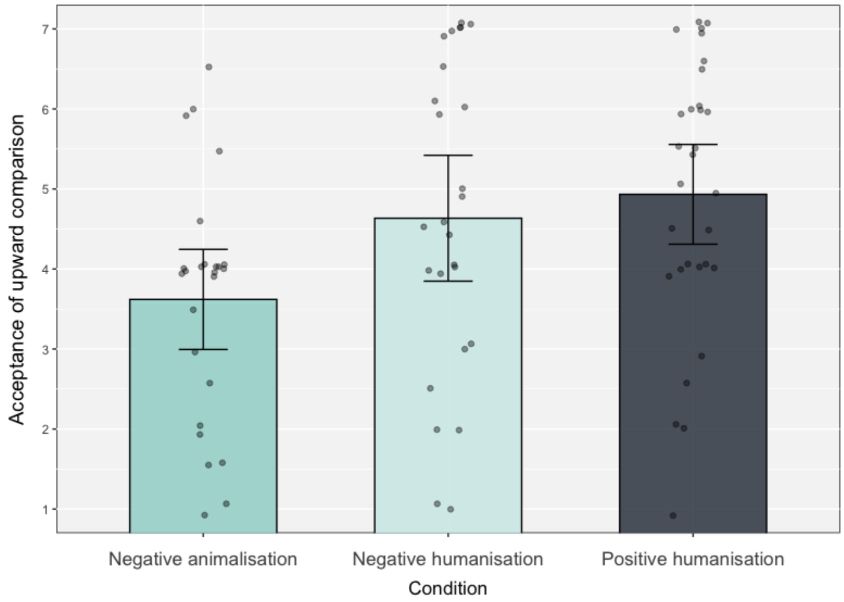

Supplementary Figure 1. Acceptance of upward comparison as a function of the experimental condition (negative animalisation, negative humanisation, positive humanisation)

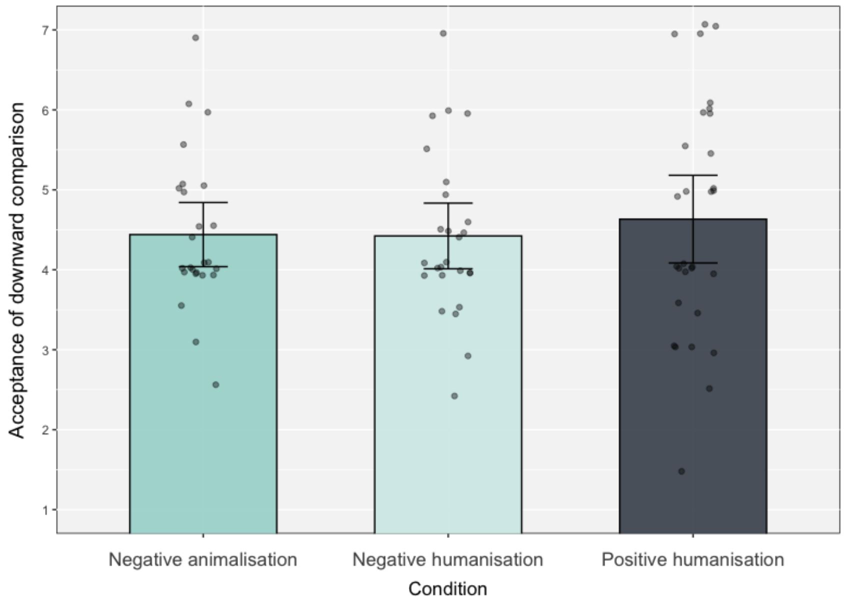

Supplementary Figure 2. Acceptance of downwards comparison as a function of the experimental condition (negative animalisation, negative humanisation, positive humanisation)

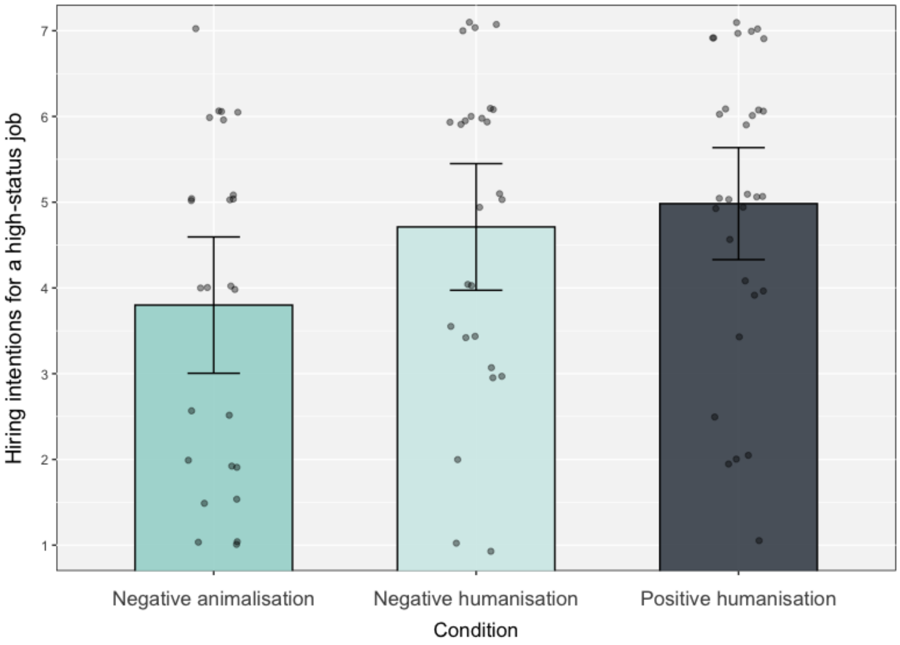

Supplementary Figure 3. Hiring intentions for a high-status job as a function of the experimental condition (negative animalisation, negative humanisation, positive humanisation)

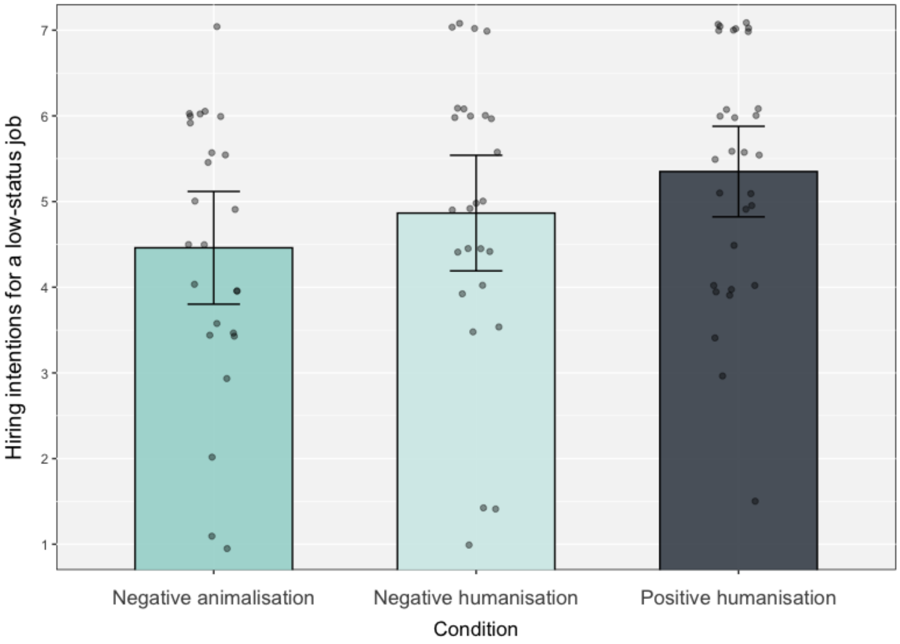

Supplementary Figure 4. Hiring intentions for a low-status job as a function of the experimental condition (negative animalisation, negative humanisation, positive humanisation)

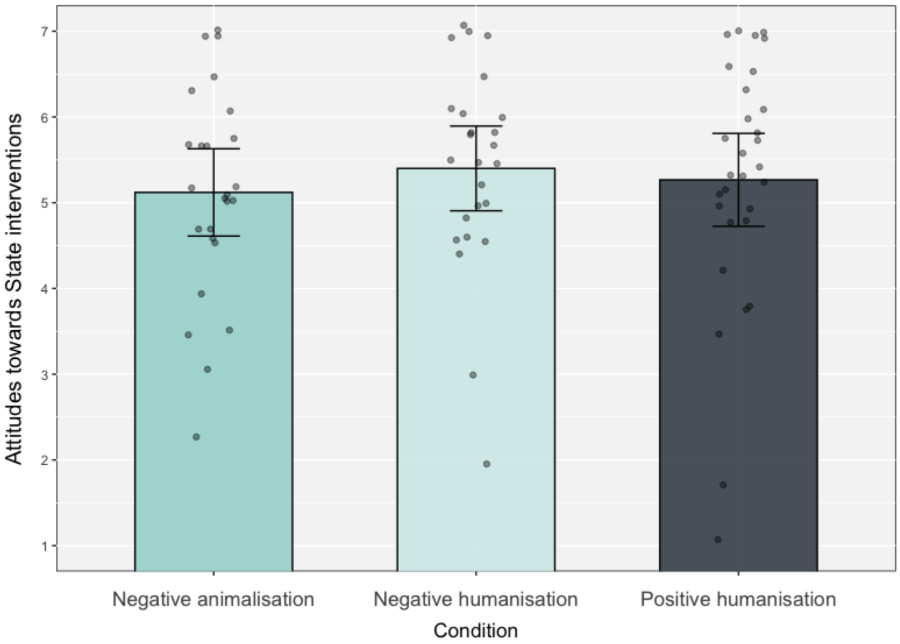

Supplementary Figure 5. Attitudes towards State interventions as a function of the experimental condition (negative animalisation, negative humanisation, positive humanisation)

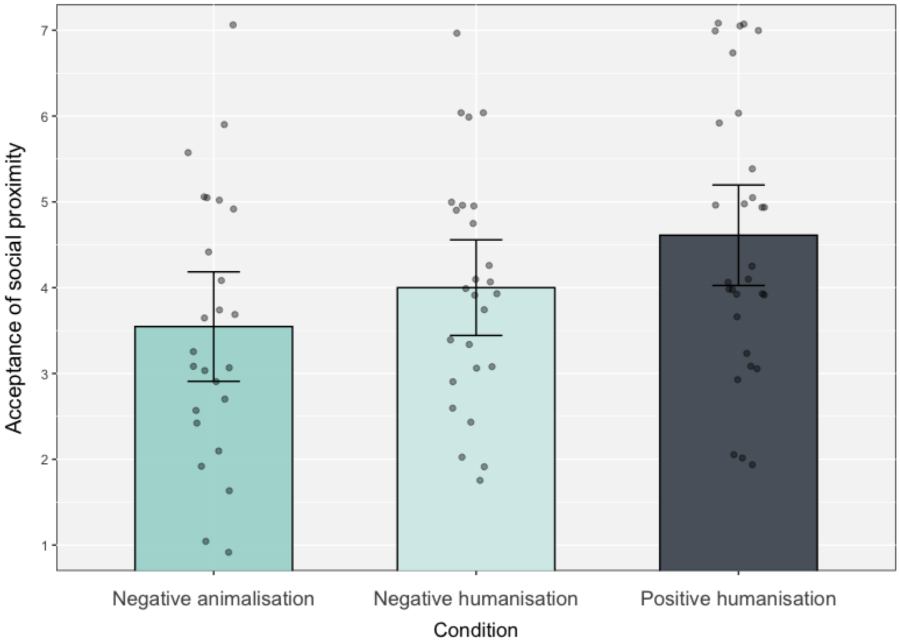

Supplementary Figure 6. Acceptance of social proximity as a function of the experimental condition (negative animalisation, negative humanisation, positive humanisation)
